# Restraint use and risky driving behaviors across drug types and drug and alcohol combinations for drivers involved in a fatal motor vehicle collision on U.S. roadways

**DOI:** 10.1186/s40621-016-0074-7

**Published:** 2016-04-01

**Authors:** Chang Liu, Yanlan Huang, Joyce C. Pressley

**Affiliations:** 1 Columbia University Mailman School of Public Health Department of Epidemiology, 722 West 168th St., Suite 812G, New York, NY 10032 USA; 2 Columbia University Department of Biostatistics, 722 West 168th St., New York, NY 10032 USA; 3 Columbia University Mailman School of Public Health Departments of Epidemiology and Health Policy and Management, 722 West 168th St., Suite 812G, New York, NY 10032 USA; 4 Center for Injury Epidemiology and Prevention at Columbia University Mailman School of Public Health, 722 West 168th St., Suite 812G, New York, NY 10032 USA

**Keywords:** Motor vehicle crash, Seatbelt use, Drugged and drunk driving

## Abstract

**Background:**

While driving impaired is a well-recognized risk factor for motor vehicle (MV) crash, recent trends in recreational drug use and abuse may pose increased threats to occupant safety. This study examines mechanisms through which drug and/or alcohol combinations contribute to fatal MV crash.

**Methods:**

The Fatality Analysis Reporting System (FARS) for 2008–2013 was used to examine drugs, alcohol, driver restraint use, driver violations/errors and other behaviors of drivers of passenger vehicles who were tested for both alcohol and drugs (*n* = 79,932). Statistical analysis was based on Chi-square tests and multivariable logistic regression. Associations of restraint use and other outcomes with alcohol and drug use were measured by estimated odds ratios (ORs) and 95 % confidence intervals (95 % CIs).

**Results:**

More than half (54.8 %) of the study population were positive for drugs or alcohol at the time of crash. Approximately half of drivers were belted, but this varied from 67.1 % (unimpaired) to 33.0 % (drugs plus alcohol). Compared to the unimpaired, the odds of a driver being unbelted varied: alcohol and cannabis (OR 3.70, 95 % CI 3.44–3.97), alcohol only (3.50,3.36–3.65), stimulants (2.13,1.91–2.38), depressants (2.09,1.89–2.31), narcotics (1.84,1.67–2.02) and cannabis only (1.55,1.43–1.67). Compared to belted drivers, unbelted drivers were over 4 times more likely to die. Driving violations varied across drug/drug alcohol combinations. Speed-related violations were higher for drivers positive for stimulants, alcohol, cannabis, and cannabis plus alcohol, with a more than two fold increase for alcohol and cannabis (2.36, 2.05, 2.71).

**Conclusions:**

Mechanisms through which drugs, alcohol and substance combinations produce increased risks to occupant safety include lowered restraint use and increases in risky driving behaviors, including speeding, lane, passing, turning and signal/sign violations.

## Background

The contribution of alcohol to motor vehicle crash risk has long been recognized (Maull et al. 1984; Soderstrom et al. 1993; Quinlan et al. 2005), but the mechanisms through which various drugs and drug combinations contribute to serious MV crash are less well understood. The prevalence of non-alcohol drug use by drivers has increased in the last few decades with marijuana becoming the leading drug detected in fatal crashes (Brady and Li 2014). With the recent trends in substance use and abuse nationally (SAMHSA 2014) and the legalization of recreational use of marijuana in several states, it has become more imperative to understand the effect of drugs on driving performance.

In laboratory and simulated driving tests, the effect of marijuana has been shown to differ between individuals, with marijuana use having a well-acknowledged effect on driving ability, including latency in reaction time and failure to keep in the proper lane (Kurzthaler et al. 1999; Ramaekers et al. 2000). The effect of stimulants on driving abilities varies among types of users with several studies demonstrating that low-dose therapeutic use of stimulants for attention deficit hyperactivity disorder patients can have a positive effect on driving abilities (Cox et al. 2000; Barkley and Cox 2007). In simulated driving experiments, high doses of stimulants have been associated with driving impairments, including increased failure to stop and signal violations (Silber et al. 2005). While other studies in Europe and Australia also suggest that the risk of causing motor vehicle crashes may differ from drug to drug (Mura et al. 2003; Drummer et al. 2004; Giovanardi et al. 2005), the mechanisms through which this occurs have not been well elucidated.

While experimental evidence obtained through simulated driving and road tests have shown that substances can have significant effects on driving performance, less is known from epidemiological studies that examine actual crash data (Gates et al. 2013; Reguly et al. 2014; Dubois et al. 2015). Recent studies have documented the increase in fatal motor vehicle crashes associated with drugged driving especially when combined with alcohol (Romano and Pollini 2013; Li et al. 2013), but these studies did not examine the distribution of driving errors and driving violations contributing to the fatality or how this might vary across drugs.

This study aims to examine the mechanisms through which this occurs, including the relationship between fatal crashes and driver characteristics, driver behavior and driving violations/errors by drug/drug category. Drug combinations are examined with and without the presence of alcohol for their association with restraint use and driving violations.

## Methods

### Data source

Data were obtained from the Fatality Analysis Reporting System (FARS) for calendar years 2008–2013. FARS is a census of all traffic fatalities on U.S. roadways. It contains person, vehicle and crash level variables. FARS captures up to three drug results for each driver. Drug detection was by means of a blood test, urine test or both. Driver characteristics (age, gender, alcohol and drug tests and violations charged), vehicle characteristics (body type), and crash characteristics (number of vehicles involved, crash-related factors, manner of collision, weekend/weekday and time of the crash) were analyzed. The data used in the analyses in this paper have been determined not human subjects research. They are all deidentified and publicly available and downloadable from National Highway Traffic Safety Administration (NHTSA) http://www.nhtsa.gov/FARS.

### Study population

**Fig. 1:**
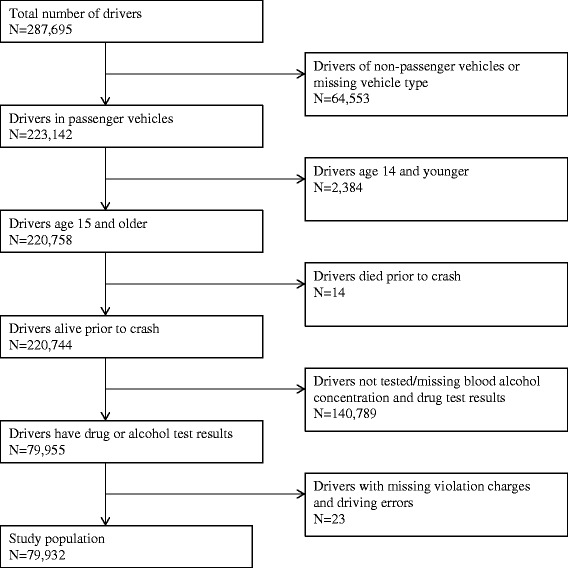
Study population

The study population was comprised of 79,932 of drivers aged 15 years and older who were tested for both alcohol and drugs after being involved in a fatal crash of a passenger vehicle. Drivers of large trucks, motor homes, motorcycles, buses, ATVs, farm equipment, large limousines, and vehicles of unknown type are excluded from analysis. Drivers with missing drug status and violation status were also excluded (Fig. 1).

### Variable definitions

#### Driver characteristics

##### Demographics

Age was categorized as 15–17, 18–19, 20–24, 25–44, 45–64 and 65 years and older. Gender was categorized as male or female as provided in FARS. Race was only coded for fatalities in FARS and was not examined in this study.

##### Restraint use

Restraint use was dichotomized: 1) not belted; and 2) belted. Shoulder belt, lap belt, shoulder and lap belt were categorized as belted.

##### Injury severity

Two variables were created to reflect injury severity. A dichotomous variable coded mortality outcome as lived or died. A second variable was categorized as: 1) not injured; 2) non-incapacitating injury; 3) incapacitating injury; and 4) fatal injury.

##### Number of occupants

Number of occupants in the vehicle was categorized into three groups: 1) driver only; 2) driver and one other passenger; and 3) driver and two or more other passengers.

##### Alcohol and drug use

FARS recorded up to three drug test results for each driver. In this study, drug/drug category was categorized into five groups: 1) narcotics; 2) depressants; 3) stimulants; 4) cannabinoid; and 5) other (phencyclidine, anabolic steroid, inhalants, and drugged, unknown type) as provided in FARS. Alcohol-involved crash was analyzed as a dichotomous variable with the driver considered positive if the blood alcohol concentration was 0.01 or higher or if police reported the driver was drinking. Among drivers considered positive for alcohol, 91.4 % had a BAC level higher than 0.05 and 84.9 % had a BAC Level higher than 0.08. Drivers with more than one type of drug/drug category or alcohol were included in the category multiple drugs/alcohol.

#### Driver violations charged/driver error

Crash related factors and driving violations were categorized as follows: sign/signal, turning, passing, reckless-driving, speed, lane or any moving violation.

##### Sign/signal violations

Violations involving red light running or other traffic signs or signals were included as a sign violation.

##### Turning violations

Violations involving improper turn, turning from the wrong lane, failure to signal intention or turn, or failure to yield were included as a turning violation.

##### Passing violations

Violations involving wrong side, passing or following too closely were included as a passing violation.

##### Reckless-driving violations

Violations involving willful and non-willful reckless, careless or hit-and-run offenses were included as a reckless violation.

##### Speed-related violation

Violations involving high speed-related offenses were categorized as a speed violation. Driving too slow was not included as a speed violation.

##### Lane violations

Violations involving improper use or erratic change of lanes were included as a lane violation as defined in FARS (FARS 2014).

##### Any moving violations

Violations involving a sign, turn, passing, reckless, speed-related or lane violations were categorized as presence of any moving violation.

##### Nonmoving violations

Violations involving validity of driver license, vehicle insurance or vehicle registrations were included as a non-moving violation.

#### Crash characteristics

##### Number of vehicles in crash

Number of vehicles in the crash was categorized as: 1) single vehicle; 2) two vehicle or 3) multiple vehicles.

##### Manner of collision

Manner of collision described the orientation of two motor vehicles in-transport and involved in a collision. Manner of collision was categorized as follows: not a collision with motor vehicle in transport, head-on, rear-end, angle, sideswipe, or other (end-swipe and others).

##### Time of crash (Day or Night)

Time of day was categorized as daytime (6:00 am to 5:59 pm) and nighttime (6:00 pm to 5:59 am).

##### Ejection

Ejection was categorized as: 1) ejected; or 2) not ejected. Partially ejected was included as ejected.

##### Weekday/weekend

Crashes occurring in the period from Friday at 6:00 pm until Monday at 5:59 am were categorized as a weekend crash.

### Statistical analysis

Bivariable descriptive analyses were performed for driver characteristics, moving violations and crash characteristics stratified by drug/drug categories. Types of moving violations were analyzed across drug/drug categories. Bivariable analyses were performed using chi-square or Fisher’s exact test. Numerical qualities were assessed for variables to be used in multivariable logistic models. The associations of different drug/drug categories, restraint use and mortality were analyzed using multivariable logistic regression with odds ratios reported with 95 % confidence intervals. Initial model selection was based on bivariable analysis with theoretical considerations and *p* ≤0.2 for variable inclusion in the model. Final models were age and gender-adjusted but otherwise included only variables whose odds ratios were significant at *p* ≤0.05. Final multivariable models were adjusted for age, gender, time of day, weekend, number of occupants. All analyses were calculated in SAS 9.4.

## Results

The study population consisted of 79,932 drivers, of which 61.1 % were aged between 25 and 64 years (Table 1). The majority of the study population was male (71.2 %). Nearly 56 % of drivers were driving solo without a passenger (Table 1). More than half (54.8 %) of the study population were positive for drugs or alcohol detected at the time of crash. Drug and alcohol use was higher in drivers aged 20–24 (23.3 %) and 24–44 (47.4 %) years. Males also accounted for the majority of drivers using drugs (67.5 %), alcohol (81.9 %) and both drugs and alcohol (79.5 %) (Table 1).

**Table 1:** Driver and crash characteristics by drug and alcohol use for drivers of passenger vehicles involved in a fatal motor vehicle crash, Fatality Analysis Reporting System (FARS), 2008–2013

	No drug *n* (%)	Drugs only *n* (%)	Alcohol only *n* (%)	Drug + Alcohol *n* (%)^a^	Total	Chi square
	36,115 (45.2)	12,771 (16.0)	18,986 (23.8)	12,060 (15.1)	79,932	
Driver characteristics						
Driver age (years)						8552.8 (<0.0001)
15–17	1764 (4.9)	411 (3.2)	273 (1.4)	247 (2.1)	2695 (3.4)	
18–19	2600 (7.2)	874 (6.8)	922 (4.9)	732 (6.1)	5128 (6.4)	
20–24	4287 (11.9)	1925 (15.1)	4106 (21.6)	2811 (23.3)	13,129 (16.4)	
25–44	9763 (27.0)	4954 (38.8)	8815 (46.4)	5673 (47.4)	29,205 (36.5)	
45–64	9806 (27.2)	3406 (26.7)	4039 (21.3)	2382 (19.8)	19,633 (24.6)	
65+	7883 (21.8)	1192 (9.3)	823 (4.3)	213 (1.8)	10,111 (12.7)	
Driver gender						2446.7 (<0.0001)
Male	23,160 (64.1)	8614 (67.5)	15,550 (81.9)	9590 (79.5)	56,914 (71.2)	
Female	12,951 (35.9)	4154 (32.5)	3430 (18.1)	2465 (20.4)	23,000 (28.8)	
Injury severity						1686.5 (<0.0001)
Not injured	5192 (15.1)	1288 (10.5)	1246 (6.8)	754 (6.5)	8480 (11.0)	
Non-incapacitating injury	2808 (8.2)	1080 (8.8)	1336 (7.3)	1067 (9.2)	6291 (8.2)	
Incapacitating injury	1954 (5.7)	1153 (9.4)	1211 (6.6)	1232 (10.6)	5550 (7.2)	
Fatal injury	24,497 (71.1)	8726 (71.3)	14,625 (79.4)	8606 (73.8)	56,454 (73.5)	
Driver restraint use						7181.9 (<0.0001)
Belted	24,212 (67.1)	6539 (51.2)	6765 (35.6)	3976 (33.0)	41,492 (51.9)	
Not belted	9616 (26.6)	1059 (40.5)	10,471 (55.2)	6878 (57.0)	32,138 (40.2)	
Number of occupants						319.7 (<0.0001)
One (Driver only)	20,030 (55.5)	7340 (57.5)	10,605 (55.9)	6535 (54.2)	44,510 (55.7)	
Two (Driver with one passenger)	6004 (16.6)	2329 (18.2)	2969 (15.6)	2324 (19.3)	13,626 (17.1)	
Three or more	3726 (10.3)	1370 (16.5)	1762 (9.3)	1423 (17.2)	8281 (10.4)	
Crash characteristics						
Violation charged						
Sign	2262 (6.3)	783 (6.1)	1084 (5.7)	817 (6.8)	4946 (6.2)	15.1 (0.0018)
Turning	1604 (4.4)	541 (4.2)	861 (4.5)	442 (3.7)	3448 (4.3)	16.2 (0.0011)
Passing	2073 (5.7)	1074 (8.4)	1809 (9.5)	1006 (8.3)	5962 (7.5)	302.7 (<0.0001)
Reckless driving	3826 (10.6)	2292 (18.0)	4376 (23.1)	3041 (25.2)	13,535 (16.9)	2134.1 (<0.0001)
Speed related	1491 (4.1)	673 (5.3)	1767 (9.3)	1142 (9.5)	5073 (6.4)	801.6 (<0.0001)
Lane	3308 (9.2)	1324 (10.4)	2522 (13.3)	1488 (12.4)	8642 (10.8)	254.3 (<0.0001)
Any moving violation	12,010 (33.3)	5393 (42.2)	9720 (51.2)	6182 (51.3)	33,305 (41.7)	2219.0 (<0.0001)
Number of vehicles in crash						6230.4 (<0.0001)
Single vehicle	13,014 (36.0)	5416 (42.4)	12,554 (66.1)	7916 (65.6)	38,900 (48.7)	
Two vehicles	18,470 (51.2)	5922 (46.4)	5311 (28.0)	3336 (27.6)	33,039 (41.3)	
Multiple vehicles	4631 (12.8)	1433 (11.2)	1121 (5.9)	808 (6.7)	7993 (10.0)	
Manner of collision						6093.8 (<0.0001)
Not a collision of vehicle in transport	14,647 (40.6)	5978 (46.8)	13,334 (70.2)	8433 (69.9)	42,392 (53.0)	
Rollover	2175 (6.0)	910 (7.1)	2177 (11.5)	1262 (10.5)	6524 (8.2)	
Tree	2266 (6.3)	1139 (8.9)	2406 (12.7)	1629 (13.5)	7440 (9.3)	
Pedestrian	2467 (6.8)	562 (4.4)	511 (2.7)	311 (2.6)	3851 (4.8)	
Other	7739 (21.4)	3367 (26.4)	8240 (43.4)	5231 (43.37)	24,577 (30.1)	
Collision of vehicle in transport	21,436 (59.4)	6785 (53.1)	5640 (29.7)	3624 (30.1)	37,485 (46.9)	
Head-on	2735 (7.6)	865 (6.8)	960 (5.1)	524 (4.3)	5084 (6.4)	
Rear-end	6346 (17.6)	2699 (21.1)	2147 (11.3)	1393 (11.6)	12,585 (15.7)	
Angle	10,754 (29.8)	2691 (21.1)	2133 (11.2)	1358 (11.3)	16,936 (21.2)	
Sideswipe	1417 (3.9)	491 (3.8)	366 (1.9)	322 (2.7)	2596 (3.3)	
Other	184 (0.5)	39 (0.3)	34 (0.2)	27 (0.2)	284 (0.4)	
Time of the crash						13,520.2 (<0.0001)
Day	22,107 (61.2)	7871 (61.6)	3866 (20.4)	2867 (23.8)	36,711 (45.9)	
Night	12,124 (33.6)	4556 (35.6)	14,624 (77.0)	8941 (74.1)	40,245 (50.4)	
Weekday/weekend						4382.8 (<0.0001)
Weekday	23,726 (65.7)	8634 (67.7)	8048 (42.4)	5499 (45.6)	45,907 (57.4)	
Weekend	11,803 (32.7)	4034 (31.6)	10,807 (56.9)	6499 (53.9)	33,143 (41.5)	
Speed						6359.1 (<0.0001)
No	29,138 (80.7)	9222 (72.2)	10,293 (54.21)	6052 (50.2)	54,705 (68.4)	
Yes	6409 (27.3)	3200 (25.1)	8196 (43.2)	5708 (47.3)	23,513 (29.4)	
Ejection						3921.4 (<0.0001)
Ejected	3956 (11.0)	2105 (16.5)	5669 (29.9)	3564 (29.6)	15,294 (19.1)	
Not ejected	32,092 (88.7)	10,628 (83.2)	13,250 (69.8)	8443 (70.0)	64,413 (80.6)	

^a^9.1 % are police reported for alcohol involvement

### Injury mortality

The majority of drivers were fatally injured (73.5 %), but this was higher in drivers with alcohol detected (79.4 %), (X^2^ = 1686.5, *p* <0.0001). Compared to drivers without drugs or alcohol, the odds ratios of fatal injury were higher for alcohol (1.99, 95 % CI: 1.90, 2.09), cannabis and alcohol in combination (1.50, 1.39, 1.61), stimulants (1.36, 95 % CI: 1.21, 1.53), depressants (1.35, 95 % CI: 1.20, 1.50), and narcotics (1.18, 95 % CI: 1.06, 1.31).

### Restraint status

Slightly more than half of drivers were belted (51.9 %), but this varied by drug and alcohol status ranging from 67.1 % belted (no drugs or alcohol detected) to 33.0 % belted (both drugs and alcohol detected) (Table 1). Belted status varied by drug/drug categories. Unbelted drivers were over 4 times more likely to die in the crash compared to belted drivers (age- and gender-adjusted odds of death, 4.42, 95 % CI: 4.25, 4.60). These are notable for drivers with both alcohol and cannabis who were 3.7 times more likely to be unbelted and drivers on alcohol were 3.5 times more likely to be unbelted. Drivers on depressants or stimulants were twice as likely to be unbelted.

### Manner of collision

Manner of collision differed among drugged and non-drugged drivers, in that, drivers with alcohol and drugs were more likely to experience rollover or a single vehicle related-crash (Table 1). Collision type was also associated with mortality, with rollover (OR = 3.17 95 % CI: 2.94, 3.42) and a tree-related crashes (OR = 5.34 95 % CI: 4.91, 5.81) having higher mortality compared to other collision types examined.

### Violations and driving errors by drug/drug category

The distribution of being charged with a moving violation varied across drug/drug category from 33.2 % for drivers without drug and alcohol detected to 51.4 % for drivers with alcohol detected (Table 2).

**Table 2:** Distribution of moving violations by drug/drug categories for drivers of passenger vehicles involved in a fatal motor vehicle crash, Fatality Analysis Reporting System (FARS), 2008–2013

	Not a moving violation *n* (%)	Any moving violation *n* (%)	Total
No drug or alcohol	22,212 (66.8)	11,047 (33.2)	33,259
Narcotics	1328 (62.5)	798 (37.5)	2126
Depressants	1159 (59.4)	793 (40.6)	1952
Stimulants	907 (53.7)	783 (46.3)	1690
Cannabis	1838 (57.0)	1385 (43.0)	3223
Alcohol	8506 (48.6)	8997 (51.4)	17,503
Cannabis+alcohol	2022 (48.9)	2112 (51.1)	4134
Any Drug	24,415 (52.3)	22,258 (47.7)	46,673

**Fig. 2:**
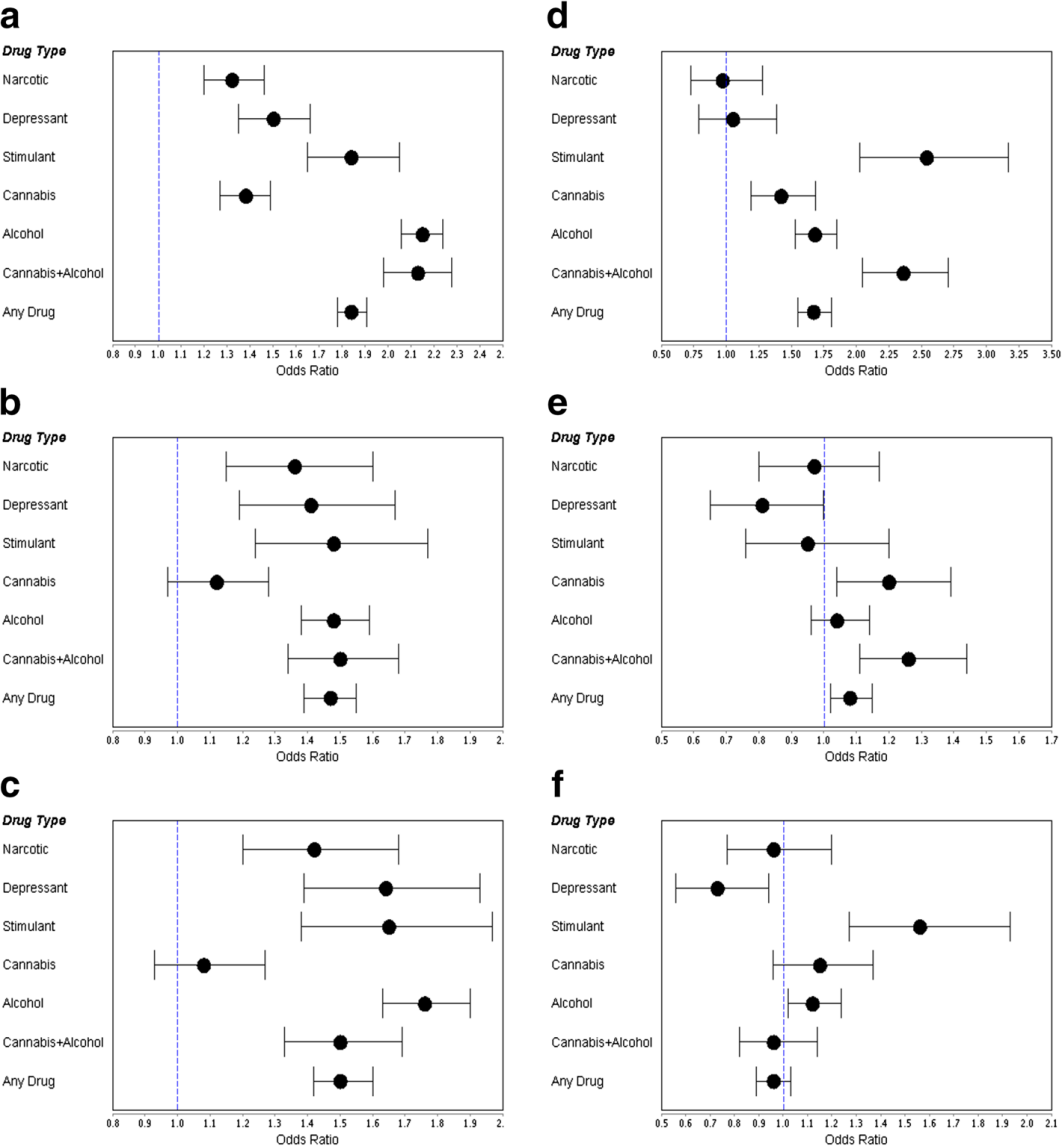
**a**–**f** Odds ratio with 95 % CI for moving violations by violation type and drug category adjusted for age, gender, time of day, weekend, number of occupants, FARS 2008–2013. **a** Total moving violations. **b** Lane violations. **c** Passing violations. **d** Speed-related violations. **e** Sign violations. **f** Turning violations

Any moving violation and reckless driving violations were increased significantly for all drug/drug categories (Fig. 2a and b). Except for cannabis, increased odds were also observed for all drug and drug categories for committing lane violations and passing violations (Fig. 2c and d). Speed-related violations were higher for drivers positive for stimulants, alcohol, cannabis, and cannabis plus alcohol (Fig. 2e). Sign violations were higher with cannabis, both with and without alcohol (Fig. 2f). Turning violations were higher with stimulants and with alcohol (Fig. 2g).

Multivariable adjusted odds of driving errors/driving violations are shown in Table 3. Drivers on narcotics or depressants exhibited similar driving patterns that differed somewhat in magnitude, with both being more likely to be cited for lane and passing violations. Drivers positive for either stimulants or alcohol, but not both, were more likely to be cited for speed-related crashes and for turning violations, in addition to the lane and passing violations seen with narcotics and depressants.

**Table 3:** Multivariable adjusted odds ratios with (95 % CI) of driving error/driving violations by drug and drug categories compared to drivers with no drug and alcohol detected^a^

	Narcotics	Depressants	Stimulants	Cannabis	Alcohol	Cannabis + Alcohol	Any drug
Driving violations							
Any moving violation	1.32 (1.12, 1.46)	1.50 (1.35, 1.66)	1.84 (1.65, 2.05)	1.38 (1.27, 1.49)	2.15 (2.06, 2.24)	2.13 (1.98, 2.28)	1.84 (1.78, 1.91)
Reckless violation	1.38 (1.21, 1.58)	1.79 (1.58, 2.0)	1.52 (1.32, 1.75)	1.55 (1.41, 1.72)	2.29 (2.17, 2.43)	2.37 (2.18, 2.57)	2.10 (2.01, 2.20)
Lane violation	1.36 (1.15, 1.60)	1.41 (1.12, 1.67)		1.48 (1.24, 1.77)	1.48 (1.38, 1.59)	1.50 (1.34, 1.68)	1.47 (1.39, 1.55)
Passing violation	1.42 (1.20, 1.68)	1.64 (1.39, 1.93)		1.65 (1.38, 1.97)	1.76 (1.63, 1.90)	1.50 (1.33, 1.69)	1.50 (1.42, 1.60)
Speed-related violation			2.54 (2.03, 3.17)	1.42 (1.19, 1.69)	1.68 (1.53, 1.85)	2.36 (2.05, 2.71)	1.67 (1.55, 1.81)
Sign violation				1.21 (1.04, 1.39)		1.11 (1.26, 1.44)	1.08 (1.02, 1.15)
Turning violation				1.56 (1.27, 1.93)		1.12 (1.02, 1.24)	0.96 (0.89, 1.03)

^a^Adjusted for age, gender, time of day, weekend, number of occupants

Drivers testing positive for either cannabis or the combination of alcohol and cannabis were more likely to be cited for sign violations, which was not significant for alcohol only impaired drivers until combined with cannabis (Table 3).

## Discussion

The major findings of this study indicate that specific drug classes and drug and alcohol combinations are associated with different patterns of driving violations among drivers involved in fatal crashes. This study extends the work of previous authors by examining and quantifying behaviors associated with fatal motor vehicle crashes including restraint use, speeding, passing and other violations across drug and drug categories (Timby et al. 1998; Longo et al. 2000).

The prevalence of non-alcohol drug use by drivers has increased in the last few years with recent studies reporting that marijuana has become the leading drug detected in fatal crashes (Brady and Li 2014). While the association of alcohol and fatal car crashes has long been recognized, the legalization of marijuana for both medical and recreational use and the presence of multiple drug combinations could pose increased threats to motor vehicle occupant safety (Anderson and Rees 2014; Johnson et al. 2012).

We also found that some categories of drug users tend to have excessive speed-related crashes, which is inconsistent with former studies (Bogstrand et al. 2015), although these studies did not examine speeding by drug/drug categories. We also found that violations and manner of collision varied by presence of drug and by drug categories.

While this study is consistent with other work that demonstrates increased mortality with drugs and alcohol, it extends these findings by describing and quantifying driving behaviors and driving violations (Mura et al. 2003; Li et al. 2013; Romano and Pollini 2013; Bédard et al. 2007). Restraint use also varied by drug and alcohol status, with the combined use of alcohol and drugs being associated with lower restraint use. Drivers on stimulants had the highest odds of being unrestrained, followed by depressants, narcotics and cannabis.

This study has limitations. First, our findings are based on drivers involved in fatal crashes who received both alcohol and drug tests. Further, drug and alcohol status is based on drug and alcohol tests, which indicate drugs detected in the body. It is not known how much the driver was under the influence of particular drug, particularly for cannabis where detection can last an extended period of time. While tetrahydrocannabinol (THC) can be detected several days after consumption of cannabis, other work in this area has noted that recent use and high doses of cannabis is a better predictor of crash risks (Ramaekers et al. 2004). We did not have this information in our data. The continual introduction of new designer drugs and substance combinations being developed may have contributed to misclassification through a decreased likelihood of drug detection and also to the presence of some drugs for which tests were not performed. To the extent this occurred, it could have resulted in lower odds of driving violations being associated with drugs. Testing also varied by several factors including crash time of day, geographic region and driver injury severity. Drivers who were fatally injured were more likely to be tested compared to those who were not fatally injured. In addition, this study used alcohol as a dichotomous variable, which didn’t control for the dose-effect of alcohol consumption on levels of impairment (Calhoun et al. 2004; West et al. 1993). However 85 % of drivers had alcohol levels that are considered legally drunk. Additional limitations of this study include that driving errors and violations are categorized based on violations charged. Earlier versions of FARS included additional information in crash-related factors, which was not available for the years examined in this study.

## Conclusions

In summary, the results of our study indicate that substance and alcohol use among drivers is associated with lower restraint use, increased driving violations and driving errors, and that these violations vary by substance and substance combinations. Our results also indicate that the presence of drug and drug and alcohol combinations exacerbate risky behaviors, such as driving unrestrained and making driving violations typically viewed as being associated with higher fatality.
